# A new species of *Neomida* Latreille from Colombia, with additional records and a complementary description for *Neomida suilla* (Champion) (Coleoptera, Tenebrionidae, Diaperini)

**DOI:** 10.3897/zookeys.495.8737

**Published:** 2015-04-08

**Authors:** Sergio Aloquio, Cristiano Lopes-Andrade

**Affiliations:** 1 Programa de Pós-Graduação em Biologia Animal, Departamento de Biologia Animal, Universidade Federal de Viçosa, 36570-900, Viçosa, MG, Brasil; 2 Departamento de Biologia Animal, Universidade Federal de Viçosa, 36570-900, Viçosa, MG, Brasil

**Keywords:** Tenebrionidae, Diaperini, *Neomida*, new species, redescription, new records, Brazil, Colombia

## Abstract

*Neomida diminuta* **sp. n.** is described, based on a single male specimen from Colombia, and a redescription of *Neomida suilla* (Champion) is given. Data on the morphology of the aedeagus for both species, and on the female abdominal terminalia for *Neomida suilla* are provided. New records of *Neomida suilla* from Atlantic Forest remnants in the states of Espírito Santo and Minas Gerais, Brazil are given.

## Introduction

Species of the genus *Neomida* Latreille, 1829 (Coleoptera: Tenebrionidae: Diaperini) are strict fungivorous beetles that dwell in hard conks of Polyporales and Hymenochaetales hosts. *Neomida* has approximately 50 described species, most from tropical and subtropical regions (Schawaller 2002). In America, the genus is most diversified in the neotropics; it does not occur in the Andean region, and has only three Nearctic species, *Neomida bicornis* (Fabricius), *Neomida occidentalis* (Champion) and *Neomida ferruginea* (LeConte). Members of *Neomida* are diagnosed by the following features (taken from Triplehorn 1965): antennal club loose and with seven antennomeres; eyes emarginate anteriorly close to antennal insertions, forming a lower portion at least twice as long as the upper portion; head of males usually bearing horns or tubercles on frons or clypeus, or both; prosternal process convex; elytral punctation seriate; basal tarsomere of hind tarsi short. However, these features are usually subject to exceptions or shared with species of other Diaperini genera. For instance, *Neomida acera* Triplehorn is devoid of secondary sexual features on male head; the long and loose antennal club of *Neomida* is similar to those of *Diaperis* Geoffroy, *Ulomoides* Blackburn and *Pentaphyllus* Dejean (Triplehorn 1965); and species of *Platydema* Laporte and Brullé also have seriate elytral punctation. *Neomida* and *Platydema* are highly diversified and use similar fungi as hosts, but can be easily distinguished at a glance: the body of *Neomida* is distinctly convex and subparallel-sided, while *Platydema* are comparatively more flattened and ovoid, with male horns on the head often asymmetric. The Neotropical fauna of *Neomida* comprises 30 described species, 16 restricted to the northern and three to the southern neotropics, and 11 species are found in both (Triplehorn 2006).

In recent field collections in southeast Brazil we found *Neomida suilla*, a species known from a few named specimens in museum collections and amongst the least studied Neotropical *Neomida*. Additionally a small undescribed *Neomida* erroneously identified as *Cis* Latreille (Ciidae) was recognized among the material borrowed from the Muséum national d’Histoire naturelle in Paris. The aims of the present work are to provide new records and a complete description for *Neomida suilla*, and describe a new species belonging to the same genus.

## Material and methods

Specimens of *Neomida suilla* were found in basidiomes of *Ganoderma* sp. (Ganodermataceae) collected in Rio Doce, in the state of Minas Gerais, and Linhares, in the state of Espírito Santo. Both localities are in the Brazilian Atlantic Forest. The beetles were reared in the laboratory, in the same fungi in which they were found, so as to obtain a high number of specimens for dissecting and depositing in scientific collections. Five adults were preserved in absolute alcohol, which are preserved below -22 °C for future molecular analyses. Forty specimens are dry mounted and several others are preserved in 70% alcohol.

**Figures 1–4.**
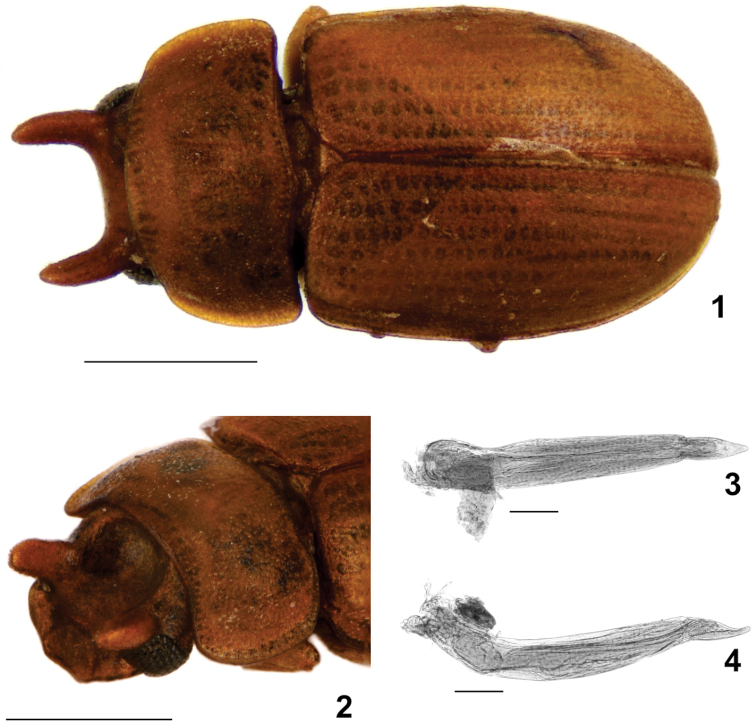
*Neomida diminuta* sp. n. male holotype. **1** Dorsal view **2** Diagonal view of head **3** Ventral view of aedeagus **4** Lateral view of aedeagus. Scales bar: 0.5 mm (Figs 1–2), 0.1 mm (Figs 3–4).

**Figures 5–9.**
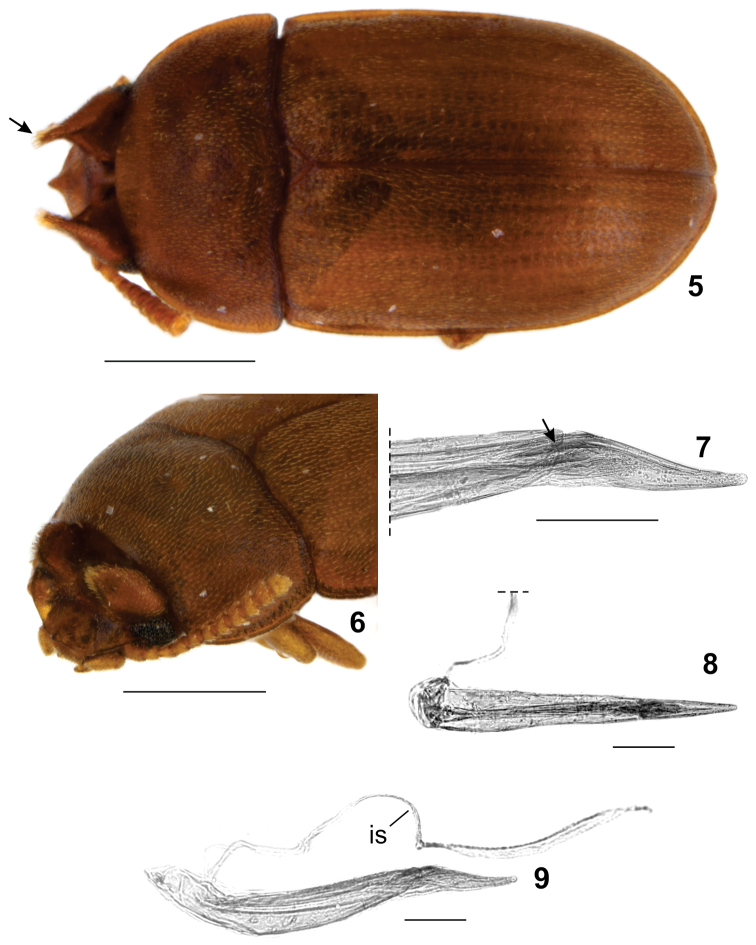
*Neomida suilla* male. **5** Dorsal view, tuft of bristles on horn tip (arrow) **6** diagonal view of head **7** Detail of aedeagus apicale showing the ala (arrow) **8** Ventral view of aedeagus **9** Lateral view of aedeagus. **is** – internal sac. Scale bars: 0.5 mm (Figs 5–6), 0.1 mm (Figs 7–9).

**Figures 10–12.**
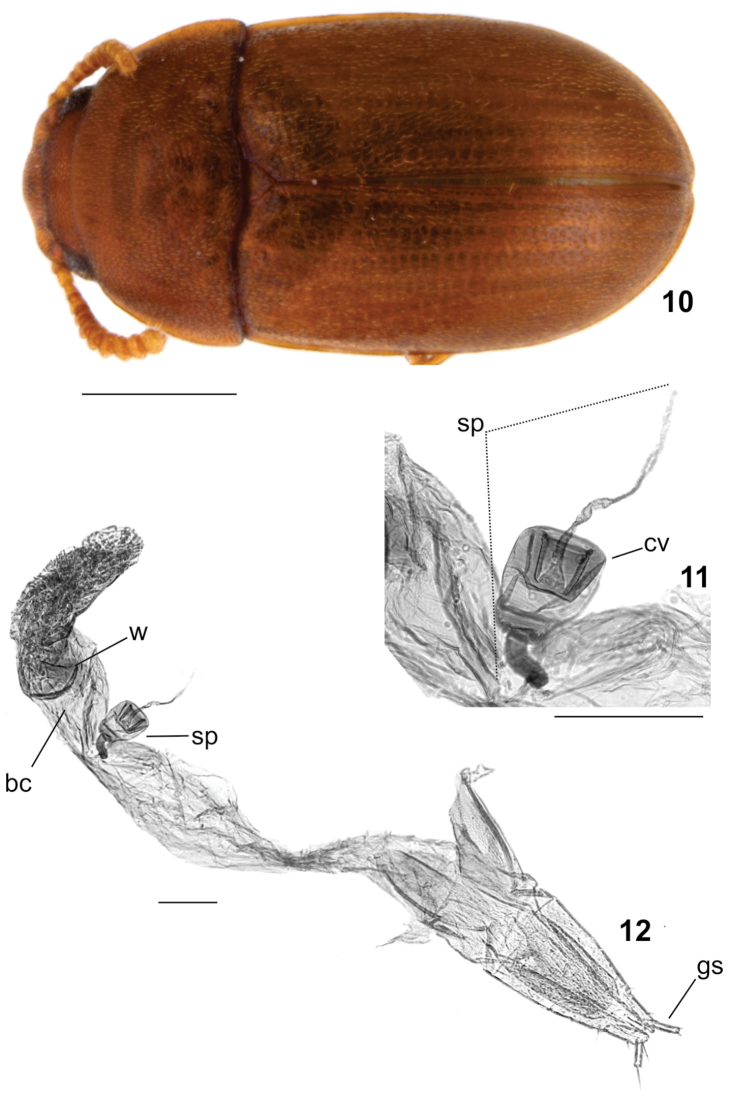
*Neomida suilla* female. **10** Dorsal view **11** Spermatheca **12** Abdominal terminalia. **bc** – bursa copulatrix, **cv** – check valve, **gs** – gonostylus, **sp** – spermatheca, **w** – window of bursa. Scale bars: 0.5 mm (Fig. 10), 0.1 mm (Figs 11–12).

Species identification was possible due to morphological data and images provided in the work of Triplehorn (2006). Specimens were examined and measured, and adult male and female abdominal terminalia extracted under a Zeiss Stemi 2000-C stereomicroscope. Female terminalia, including spermatheca, were stained with a solution of 0.5% Chlorazol Black E in 85% alcohol to enhance contrast. Whole mount preparations of dissected sclerites were made using a water-soluble mounting media based on polyvinyl alcohol and lactic acid. We photographed slides under a Zeiss AxioLab compound microscope equipped with a Zeiss AxioCam ERc 5s digital camera (Figs 3–4) and a Zeiss AxioCam MRc (Figs 7–9, 11–12), and adult specimens under a Zeiss Discovery V8 stereomicroscope with a Zeiss AxioCam MRc digital camera (Figs 1–2) and a Zeiss Discovery V20 stereomicroscope with a Zeiss AxioCam 506 (Figs 5–6, 10). Final images were the result of montaging 25 to 125 image slices at different focal lengths using the extended focus module of Zeiss Axiovision 4.8 software (Figs 1–2) and Zeiss ZEN 2012 (Figs 5–6, 10).

We based the redescription of *Neomida suilla* on a male plesiotype (a specimen used for a redescription, supplementary description, or illustration published subsequent to the original description; sensu Evenhuis 2008), and the description of *Neomida diminuta* sp. n. on a single male from Colombia. Terms for external morphology, including sclerites of abdominal terminalia, follow Lawrence et al. (2011). The term basale refers to the phallobase, and apicale to the fused parameres (Lawrence et al. 2011). The following symbols are used for measurements (in mm) and ratios: EL, elytral length (at midline, from base of scutellum to elytral apex); EW, greatest elytral width; GD, greatest depth of the body (from elytra to metaventrite); PL, pronotal length along midline; PW, greatest pronotal width; TL, total length (=EL+PL; head not included). The ratio GD/EW was recorded as an indication of degree of convexity; TL/EW indicates degree of body elongation.

**Figure 13.**
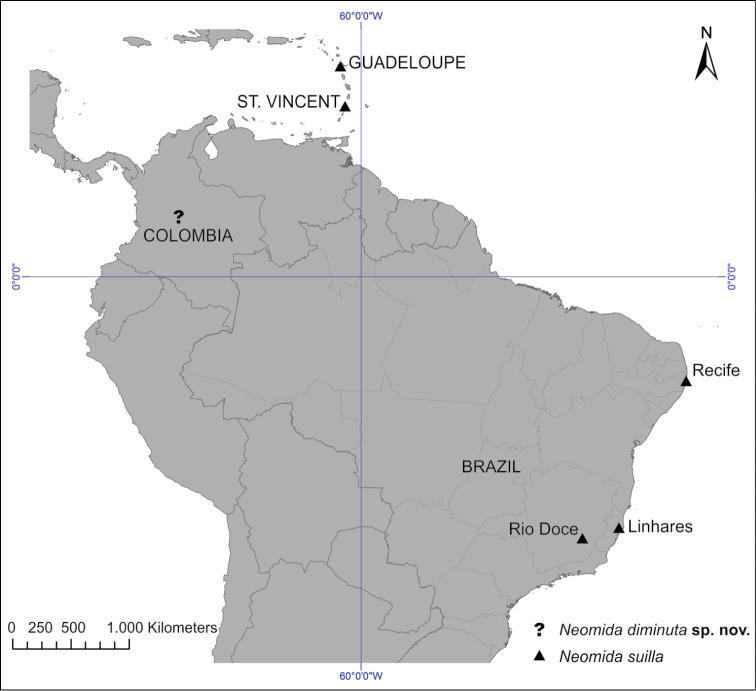
Distribution map for *Neomida diminuta* sp. n., represented by an interrogation symbol (?), without specific locality, and *Neomida suilla*, represented by a triangle (▲).

The distribution map (Fig. 13) was created using latitude and longitude coordinates estimated by tracking localities in the online database GeoNames (Wick 2010) and plotting them in a map using DIVA-Gis 7.5.

Labels were printed in white paper, unless otherwise specified. Label data are cited verbatim in quotation marks; a backslash separates different labels. Square brackets are used to denote our comments on label data. The number and gender of specimens bearing these labels are stated immediately before the label data.

### Acronyms of depositories

ANIC Australian National Insect Collection, CSIRO Ecosystem Sciences (Canberra, Australia)

CELC Coleção Entomológica do Laboratório de Sistemática e Biologia de Coleoptera, Universidade Federal de Viçosa (Viçosa, Minas Gerais, Brasil)

MNHN Muséum national d’Histoire naturelle (Paris, France)

OSUC The Ohio State University Insect Collection (Columbus, Ohio, USA)

## Taxonomy

### Neomida diminuta sp. n.

Taxon classificationAnimaliaColeopteraTenebrionidae

http://zoobank.org/74A4E927-BEA1-4763-857E-20A3899F26B1

Figs 1–4

#### Diagnosis.

*Neomida diminuta* sp. n. differs from all other *Neomida* by its minute size (TL 1.74 mm), while other species in the genus are at least 1.85 mm long. It differs from *Neomida suilla* and *Neomida picea* in the possession of two clypeal tubercles instead of one, from *Neomida cioides* in the subcylindrical and straight frontal horns, and from *Neomida inermis* by its subtle clypeal sinuosity instead of conspicuous and cylindrical tubercles. *Neomida diminuta* sp. n. males have eyes, body shape and cephalic horns similar to those of *Neomida occidentalis*, but the latter are twice as long. In *Neomida diminuta* sp. n., the epipleura extends from base to apex of elytra, a feature observed only in other six species of *Neomida*: *Neomida cioides* (Champion), *Neomida deltocera* Triplehorn, *Neomida occidentalis*, *Neomida pentaphyllodes* (Champion), *Neomida picea* (Laporte and Brullé) and *Neomida suilla*.

#### Etymology.

The name “diminuta” means small, referring to its minute size.

#### Description.

**Male. Body** moderately convex, opaque, glabrous; length 1.74 mm; elytra, pronotum and head reddish-brown; antennae and legs golden-yellow. **Head** with vertex deeply concave; frons armed with a pair of long, subcylindrical, subparallel narrow horns, each rising close to an eye; clypeus with two small sinuosities contiguous to antennal insertions. **Eyes** with anterior portion emarginated by antennal insertion, forming a lower lobe approx. four times as large as upper lobe. **Antennae** with antennomeres 5–11 expanded forming a club. **Pronotum** strongly transverse, approx. twice as wide as long, widest and longest at middle, sides subparallel and narrowed anteriorly; lateral edges explanate, visible for their entire lengths from above; anterior edge truncate. **Elytra** approx. twice as long as pronotum, widest at middle and narrowing to apex, epipleura extending to apex. **Hind wings** developed, apparently functional. **Ventral surface** slightly darker than dorsum, punctation sparser; prosternal process subparallel. **Protibiae** with outer edge serrate; apex bearing a row of spines; inner apical angle with two long spines. **Hind tarsi** with basal tarsomere approx. as long as the following three together. **Aedeagus** with basale approx. three and a half times as long as apicale, curved at base, sides subparallel, a bit wider in the second third; apicale with sides subparallel, narrowing near middle to apex; penis about as long as basale, cylindrical, expanded at apex, with struts converging and fusing at basal one-fifth; internal sac not observed (possibly lost during dissection). **Female** unknown.

#### Measurements.

Male holotype (in mm): TL 1.74, PL 0.49, PW 0.89, EL 1.17, EW 0.98, GD 0.69; ratios: PL/PW 0.55, EL/EW 1.19, EL/PL 2.39, GD/EW 1.70, TL/EW 2.46.

#### Type specimen.

Male holotype (MNHN) labeled: “Dup Colomb 41 [sic] {circular green label}\ ♂ {small green label}\ *Neomida diminuta*, HOLOTYPUS, Aloquio & Lopes-Andrade {handwritten in red label}”.

#### Comments.

*Neomida diminuta* sp. n. was collected in 1841 and remained unrecognized as a tenebrionid beetle in the Muséum national d’Histoire naturelle of Paris, France, until recently. It was found among specimens identified as *Cis* Latreille (Ciidae), possibly confounded due to its small size and head bearing horns. The great age certainly affected important morphological features, such as body vestiture, pronotal and elytral punctation and integrity of membranous structures as the internal sac of aedeagus. Information on host fungus was not available. The extension of exposed epipleura and other conspicuous characters need to be more carefully observed, because they can be important for proposing species-groups or even subgenera for *Neomida*, in order to facilitate the work with such a speciose genus.

### Neomida suilla

Taxon classificationAnimaliaColeopteraTenebrionidae

(Champion, 1896)

Figs 5–9 , 10–12

Arrhenoplita suilla Champion 1896: 11
Hoplocephala suilla (Champion): Blackwelder 1945: 527
Neomida suilla (Champion): Triplehorn 1965: 375; Marcuzzi 1984: 87; Triplehorn 2006: 313

#### Diagnosis.

Males of *Neomida suilla* differ from males of all other described New World *Neomida*, except *Neomida picea* (Laporte and Brullé), in having a single prominent median clypeal tubercle (Triplehorn 2006). *Neomida suilla* differs from *Neomida picea* by its shorter length, and males in having triangular-shaped cephalic horns with a tuft of bristles at their tips (Fig. 5, arrow).

#### Supplementary description.

**Male. Body** moderately convex, opaque, with vestiture of small seta; length 1.85–2.25 mm; elytra, pronotum and head reddish-brown; antennae, legs and mouthparts golden-yellow. **Head** with clypeus bearing a single prominent tubercle near the middle of anterior edge; frons armed with a pair of long, flattened, subtriangular, broad horns, each rising close to an eye and directed upward; horns with a row of bristles extending from about the middle of anterior edge to apex (Fig. 5, arrow); vertex deeply concave; **Eyes** with anterior portion emarginate by antennal insertion, forming a lower lobe about three times larger than upper lobe. **Antennae** with antennomeres 5–11 expanded forming a club; antennomeres 6–11 bearing multi-pronged sensilla (sensillifers) at the upper portion. **Pronotum** strongly transverse, twice as wide as long, widest posteriorly and longest at middle; lateral edges explanate, visible for their entire lengths from above; anterior edge slightly curved outward. **Elytra** approx. two and a half times as long as pronotum; sides subparallel at basal half, then narrowing to apex, epipleura extending to apex. **Hind wings** developed, apparently functional. **Ventral surface** slightly darker than dorsum, punctation sparser; prosternal process subparallel, narrowest at apex. **Protibiae** with outer edge serrate; apex bearing a row of spines; inner apical angle with two long spines. **Aedeagus** with basale about three times as long as apicale; basale most expanded near its base; apicale strongly narrowed at apex and with two lateral projections (ala) directed anteriorly and fitting the basale (Fig. 7, arrow); penis about as long as basale, cylindrical, expanded at apex, with struts converging and fusing at basal one-seventh of the length; internal sac narrow, elongated, about twice as long as penis. **Females** similar to males except for the following features: head without clypeal tubercle and frontal horns, and vertex devoid of concavity; eyes with lower lobe twice as long as upper one. **Female abdominal terminalia** with bursa copulatrix approx. one and a half time as long as gonocoxites together; common oviduct approx. as long as window of bursa; window of bursa about four times as long as spermatheca; spermatheca (Fig. 11) with check valve small, oval, bearing an invagination from upper portion to about middle; paraprocts about as long as gonocoxites together; baculi of basal gonocoxites perpendicular in relation to baculi of paraprocts; gonocoxites transversely divided into three parts; gonostyli inserted at top of apical gonocoxites.

#### Variation.

Males (n = 30), measurements (in mm): TL 1.90–2.20 (2.10 ± 0.09), PL 0.45–0.60 (0.56 + 0.04), PW 0.90–1.05 (1.00 + 0.04), EL 1.00–1.40 (1.28 + 0.08), EW 0.95–1.10 (1.05 + 0.04), GD 0.70–0.80 (0.74 + 0.03); ratios: PL/PW 0.50–0.60, EL/EW 1.00–1.35, EL/PL 2.00–2.67, GD/EW 0.64–0.75, TL/EW 1.82–2.15. Females (n = 10), measurements (in mm): TL 1.85–2.20 (2.08 + 0.10), PL 0.45–0.60 (0.56 + 0.05), PW 0.90–1.05 (1.00 + 0.04), EL 1.25–1.40 (1.32 + 0.05), EW 0.95–1.10 (1.06 + 0.05), GD 0.70–0.75 (0.74 + 0.02); ratios: PL/PW 0.50–0.60, EL/EW 1.18–1.35, EL/PL 2.17–2.78, GD/EW 0.68–0.74, TL/EW 1.91–2.10.

#### Material examined.

15 males and five females (1♂ and 1♀ ANIC, 13♂ and 3♀ CELC, 1♂ and 1♀ OSUC) labeled: “BRASIL: MG, Rio Doce, Lago da Candonga; área de mata, 16.ix.2009, leg. E.F. Barbosa”. 15 males and five females (1♂ and 1♀ ANIC, 13♂ and 3♀ CELC, 1♂ and 1♀ OSUC) labeled: “BRASIL: ES, Linhares, Mata do Lago, 16.vii.2010, leg. S.Z. Aloquio Jr.”.

#### Comments.

All specimens of *Neomida suilla* were found in basidiomes of *Ganoderma* sp., which is the primary host fungus record for the species. *Neomida suilla* was collected in two localities of the Brazilian Atlantic Forest (Fig. 13) separated by approx. 500 linear km, each at an extreme of the Doce River. These two localities are the most southern records for the species, which was known from only two localities in the Lesser Antilles (St. Vincent and Guadeloupe) and one in Recife (in the state of Pernambuco, Brazil). Its occurrence in the Lesser Antilles and in distant localities of the Brazilian Atlantic Forest suggests a wide distribution throughout the east coast of tropical South America.

## Supplementary Material

XML Treatment for Neomida diminuta

XML Treatment for Neomida suilla
